# IL-2 in the tumor microenvironment is necessary for Wiskott-Aldrich syndrome protein deficient NK cells to respond to tumors *in vivo*

**DOI:** 10.1038/srep30636

**Published:** 2016-08-01

**Authors:** Joanna S. Kritikou, Carin I. M. Dahlberg, Marisa A. P. Baptista, Arnika K. Wagner, Pinaki P. Banerjee, Lavesh Amar Gwalani, Cecilia Poli, Sudeepta K. Panda, Klas Kärre, Susan M. Kaech, Fredrik Wermeling, John Andersson, Jordan S. Orange, Hanna Brauner, Lisa S. Westerberg

**Affiliations:** 1 Department of Microbiology Tumor and Cell biology, Karolinska Institutet, Stockholm 171 77, Sweden; 2 Center for Human Immunobiology, Baylor College of Medicine and Texas Children’s Hospital, Houston, TX 77030, USA; 3 Department of Medicine Solna, Karolinska Institutet, and Karolinska University Hospital, Stockholm 171 76, Sweden; 4 Department of Immunobiology, Yale University School of Medicine, New Haven, CT 06520, USA; 5 Howard Hughes Medical Institute, 4000 Jones Bridge Road, Chevy Chase, MD 20815, USA

## Abstract

To kill target cells, natural killer (NK) cells organize signaling from activating and inhibitory receptors to form a lytic synapse. Wiskott-Aldrich syndrome (WAS) patients have loss-of-function mutations in the actin regulator WASp and suffer from immunodeficiency with increased risk to develop lymphoreticular malignancies. NK cells from WAS patients fail to form lytic synapses, however, the functional outcome *in vivo* remains unknown. Here, we show that WASp KO NK cells had decreased capacity to degranulate and produce IFNγ upon NKp46 stimulation and this was associated with reduced capacity to kill MHC class I-deficient hematopoietic grafts. Pre-treatment of WASp KO NK cells with IL-2 *ex vivo* restored degranulation, IFNγ production, and killing of MHC class I negative hematopoietic grafts. Moreover, WASp KO mice controlled growth of A20 lymphoma cells that naturally produced IL-2. WASp KO NK cells showed increased expression of DNAM-1, LAG-3, and KLRG1, all receptors associated with cellular exhaustion and NK cell memory. NK cells isolated from WAS patient spleen cells showed increased expression of DNAM-1 and had low to negative expression of CD56, a phenotype associated with NK cells exhaustion. Finally, in a cohort of neuroblastoma patients we identified a strong correlation between WASp, IL-2, and patient survival.

Natural killer (NK) cells eliminate virus-infected cells and cancer cells. NK cell mediated killing occurs when inhibition is lost because the target cell lacks one or more self MHC class I molecules (“missing self”) or when target cells have high expression of stimulatory ligands and produce cytokines that override inhibition^1^,^2^,^3^,^4^,^5^,^6^. NK cells express a repertoire of activating and inhibitory receptors and the balance in signaling between these receptors determines the outcome of the NK cell response. NK cells develop in the bone marrow, where they start to express Ly49 receptors that enable recognition of MHC class I^7^. Moreover, NK cells undergo education to ensure that only the NK cells that can be inhibited by self MHC class I molecules become functional competent killer cells^7^,^8^,^9^.

NK cells express receptors that regulate co-stimulation and are associated with cellular exhaustion of T cells and NK cells^10^. Cytotoxic T lymphocyte antigen 4 (CTLA-4) binds with high affinity to CD80/CD86 and prevents co-stimulation^10^. Programmed cell death protein 1 (PD-1) has upon binding to the ligands PD-L1 and PD-L2 the capacity to suppress transcription of specific genes^10^. Lymphocyte-activation gene 3 (LAG-3) shares homology to CD4 and binds to MHC class II^11^. Inhibitory Killer cell lectin-like receptor G1 (KLRG1) binds to E-, N-, and R-cadherins on target cells and is expressed on the most mature NK cells^12^,^13^. Recent data suggests that mature NK cells that express KLRG1 are the most efficient killer cells^14^.

NK cells integrate signals from the environment by forming two types of immunological synapses; one inhibitory synapse mediated by inhibitory receptors and one activating lytic synapse meditated by activating receptors^15^. NK cells from Wiskott-Aldrich syndrome (WAS) patients have decreased polarization of actin, MTOC, and lytic vesicles in the synapse interface to target cells^16^,^17^. The tumor incidence in WAS is estimated to be 13–22% with a poor prognosis and most frequently associated with lymphoreticular tumors including non-Hodgkin lymphoma (76% of the total tumors associated with WAS), Hodgkin disease, and Burkitt lymphoma^18^,^19^,^20^,^21^,^22^.

WASp knockout (KO) mice bred with tumor-prone mice have accelerated onset of tumor growth and B16 melanoma cells are more metastatic in WASp KO mice^23^. In another study, breast carcinoma cells had similar tumor growth in WT and WASp KO mice^24^, however, WASp KO mice had decreased metastatic spread^24^. Thus, the data from these two studies are somewhat contradictory and the extent of WASp KO NK cell dysfunction may depend on the tumor context. Importantly, the cytolytic defect of WAS patient NK cells can be rescued by addition of exogenous IL-2^17^,^25^ that induces phosphorylation of WAVE2 and actin polymerization^17^. This has prompted initiation of clinical trials for IL-2 treatment of WAS patients as described for the first treated patient^17^. The efficacy of IL-2 treatment in WASp deficiency relies on that NK cells develop normally, are educated correctly, and that they are responsive to IL-2 treatment *in vivo*. Moreover, the outcome of IL-2 treatment for NK cell-mediated killing of lymphoreticular tumors, including lymphomas that is a major cause of death in WAS patients, remains unknown.

Tumorigenesis is difficult to study in WAS patients since it develops late in life and the vast majority of patients rapidly undergo hematopoietic stem cell transplantation today. We took the approach to investigate tumorigenesis in WASp knockout (KO) animals. We found that WASp KO NK cells developed normally but had increased expression of DNAM-1, KLRG1, and LAG-3. WASp KO NK cells had decreased formation of immune synapses with lymphoma target cells and reduced killing capacity of MHC class I negative hematopoietic grafts *in vivo*. We identify that IL-2, either exogenously provided or produced naturally by lymphoma cells, restored killing capacity by WASp KO NK cells *in vivo*. NK cells isolated from WAS patient spleen cells showed increased expression of DNAM-1 and had low to negative expression of CD56, a phenotype associated with NK cells exhaustion. Finally, in cohorts of neuroblastoma patients and patients with diffuse large B cell lymphoma we identified a strong correlation between WASp, IL-2, and patient survival.

## Results

### Reduced rejection of MHC class I- hematopoietic grafts in WASp KO NK mice

**Figure 1:**
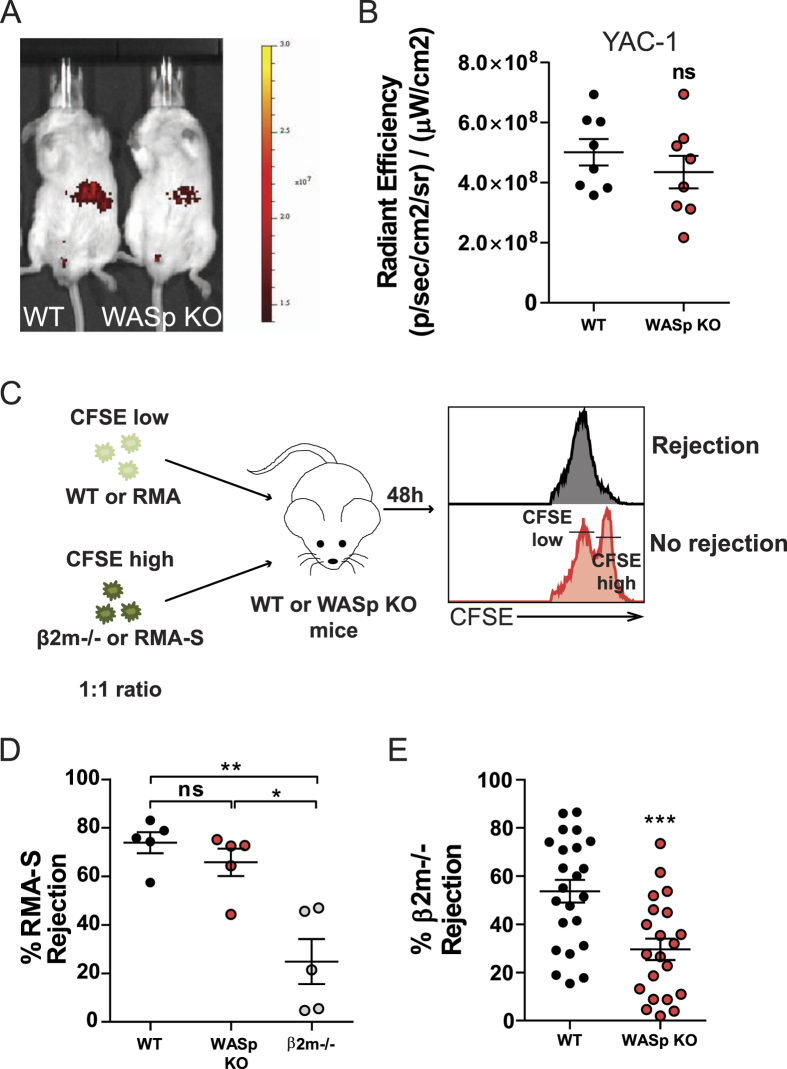
Reduced rejection of MHC class I^−^ hematopoietic grafts in WASp KO NK mice. (**A**) *In vivo* imaging of Balb/c mice injected with 1 × 10^6^ YAC-1 lymphoma cells. Cells were labelled with the DiR dye to assess tumor growth, as a measure of fluorescence. Scale: Radiant efficiency in (p/sec/cm^2^/sr)/(mW/cm^2^). (**B**) YAC-1 tumor growth in Balb/c mice using *in vivo* imaging after 5h of injection plotted as radiant efficiency, from a pool of 3 individual experiments. Autofluorescence measured in a non-injected mouse was subtracted from injected mice at 5 hr. WT n = 8, WASp KO n = 8. (**C**) *In vivo* rejection protocol in C57BL/6 mice. MHC class I deficient splenocytes (β2m^−/−^) or lymphoma cells (RMA-S) were labelled with a high CFSE concentration. WT splenocytes or RMA lymphoma cells were labelled with a low CFSE concentration. A 1:1 cell mix is then injected into WT or WASp KO mice and rejection is measured after 2 days. (**D**) *In vivo* lymphoma cell rejection assay in C57BL/6 mice shown as percentage of rejected RMA-S cells, from a pool of 2 individual experiments. WT n = 5, WASp KO n = 5, β2m^−/−^ n = 5. (**E**) *In vivo* splenocyte rejection assay in C57BL/6 mice shown as percentage of rejected β2m^−/−^ cells, from a pool of 2 individual experiments. WT n = 10, WASp KO n = 10. Outliers based on ROUT (Q = 1%) excluded: WT n = 1.Each dot represents one mouse. Graphs show mean values ± SEM. Significance was assessed with the Mann-Whitney test. **P < 01, ***P < 001 and ns  =  not significant.

To examine the tumor rejection capacity in WASp KO mice, allogeneic YAC-1 T cell lymphoma cells were injected intravenously and monitored at 5 h by *in vivo* imaging (IVIS). WT and WASp KO mice showed similar growth of YAC-1 cells (Fig. 1A,B). To address the role of NK cell-mediated tumor rejection in WASp KO mice, we performed a competitive assay in which we injected T cell lymphoma cells expressing MHC class I (RMA) or with reduced expression of MHC class I (TAP^−/−^; RMA-S), labeled with different concentrations of CFSE (Fig. 1C). Both wildtype and WASp KO C57Bl/6 mice could efficiently reject RMA-S T cell lymphoma cells (Fig. 1D). We next performed the competitive assay using wildtype syngenic splenocytes together with syngenic MHC class I negative (β2m^−/−^) splenocytes, labeled with different concentrations of CFSE (Fig. 1C). In wildtype mice, β2m^−/−^ splenocytes will be rejected by NK cells due to missing-self recognition. While up to 80% of β2m^−/−^ cells were rejected in wildtype mice, WASp KO mice had decreased capacity to reject β2m^−/−^ cells (Fig. 1E). This data suggested that WASp KO NK cells failed to reject hematopoietic grafts lacking MHC class I molecules. However, if the tumor cells express multiple activating ligands and/or cytokines, such as YAC-1 and RMA-S lymphoma cells, WASp KO mice controlled tumor growth similar to wildtype mice.

### WASp KO NK cells develop and are educated normally

**Figure 2:**
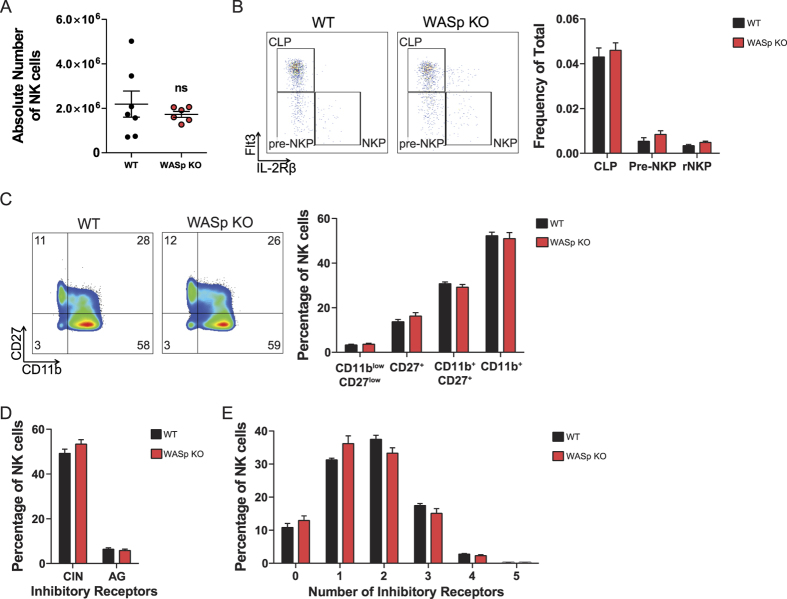
WASp KO NK cells develop and are educated normally. (**A**) NK cell number. The absolute number of NK1.1^+^CD3^−^ NK cells in the spleen of WT and WASp KO mice, from a pool of 2 individual experiments. Each dot represents one mouse. WT n = 7, WASp KO n = 6. (**B**) NK cell development in the bone marrow. CD19^−^CD11b^−^Ly6D^−^NK1.1^−^CD3^−^c-Kit^−^2B4^+^CD27^+^IL-7Rα^+^ bone marrow cells were used to define the 3 different NK cell precursor populations; CLP, Pre-NKP and NKP cells, based on Flt3 and IL-2Rβ expression. The analysis is a pool of 2 individual experiments. WT n = 6, WASp KO n = 6. (**C**) NK cell development assessed with markers CD11b and CD27. The analysis is a pool of 3 individual experiments. WT n = 10, WASp KO n = 11. (**D**,**E**) Analysis of the inhibitory receptor repertoire to assess NK cell education in WASp KO mice, from a pool of 2 individual experiments. WT n = 4, WASp KO n = 6. (**D**) The number of NK cell inhibitory receptors reacting to self or non-self MHC class I in C57Bl/6 hosts. The two classes of inhibitory receptors; CIN = Ly49C, Ly49I and/or NKG2A, AG  =  Ly49A and/or Ly49G2. NK cells that express any of the combinations between both group CIN and AG (C + A, C + G, I + A, I + G, N + A, N + G, CI + A, CI + G, IN + A, IN + G, CN + A, CN + G, CI + AG, IN + AG, and CN + AG) are not shown. (**E**) The number of different inhibitory receptors per NK cells. Graphs show mean values ± SEM. Significance was assessed with the Mann-Whitney test and found not significant when comparing WT and WASp KO values. ns  =  not significant. Abbreviations: CLP; Common Lymphoid Progenitor, Pre-NKP; Pre-NK cell Progenitor, NKP; NK cell progenitor.

The observed defect in WASp KO NK cell killing of MHC class I negative hematopoietic grafts cells could be caused by aberrant education and/or development. Wildtype and WASp KO mice had similar number of NK cells in the spleen (Fig. 2A and supplementary Fig. 1A) and both mice had normal frequency of NK cell progenitor cells in the bone marrow, including common lymphoid progenitor (CLP) cells, pre-NK progenitor cells (pre-NKP), and NK cell progenitor cells (NKP, Fig. 2B and supplementary Fig. 1B)^26^. Moreover, WASp KO NK cells developed normally in the spleen as examined by profiles for the final maturation stages; CD27^−^CD11b^−^, CD27^+^CD11b^−^, CD27^+^CD11b^+^, and CD27^−^CD11b^+^ NK cells (Fig. 2C). The inhibitory receptors expressed on the surface of NK cells educate them to recognize self MHC class I molecules and inhibit NK cell activation during an effector phase, when self MHC class I molecules are expressed. By determining the expression of self specific and non-self specific inhibitory receptors on NK cells, education can be assessed. We first examined the frequency of NK cells expressing self-MHC-I-specific inhibitory receptors Ly49C (C), Ly49I (I) or NKG2A (N) or any combination of the three (C + I + N + CI + CN + IN + CIN) versus NK cells expressing non-self-specific inhibitory receptors Ly49A (A) or Ly49G2 (G) or any combination thereof (A + G + AG). CIN^+^ NK cells are educated in mice expressing K^b^D^b^ MHC class I molecules such as C57BL/6 mice whereas AG^+^ NK cells do not encounter a strong MHC-I ligand in mice of the C57BL/6 background and are therefore considered to be non-educated. A high proportion of NK cells (50%) from wildtype and WASp KO mice expressed the inhibitory receptors Ly49C, Ly49I, and NKG2A that recognize self H-2^b^ MHC class I molecules in C57BL/6 mice (CIN, Fig. 2D and supplementary Fig. 1C). Less than 10% of the NK cells from both wildtype and WASp KO mice expressed any of the non-self specific inhibitory receptors Ly49A and Ly49G2 that lack MHC class I ligand in C57BL/6 hosts (AG, Fig. 2D). One of the key features of an educated NK cell receptor repertoire in MHC class I^+/+^ mice is a shift towards a repertoire where many NK cells express only few (1–2) inhibitory receptors, while in MHC class I^−/−^ mice a lot of NK cells express many (3–5) inhibitory receptors^27^. This phenomenon can be explained by the fact that an NK cell with only 1 or 2 inhibitory receptors is actually the most competent one in protecting against transformation or infection. Wildtype and WASp KO NK cells had similar frequency of cells expressing 1–2 inhibitory receptors (Fig. 2E), suggesting that WASp KO NK cells were educated correctly *in vivo* with regards to inhibitory receptor expression. Together, this data suggests that WASp KO NK cells had normal development and education.

### WASp KO NK cells have increased CD69, DNAM-1, LAG-3, and KLRG1

**Figure 3:**
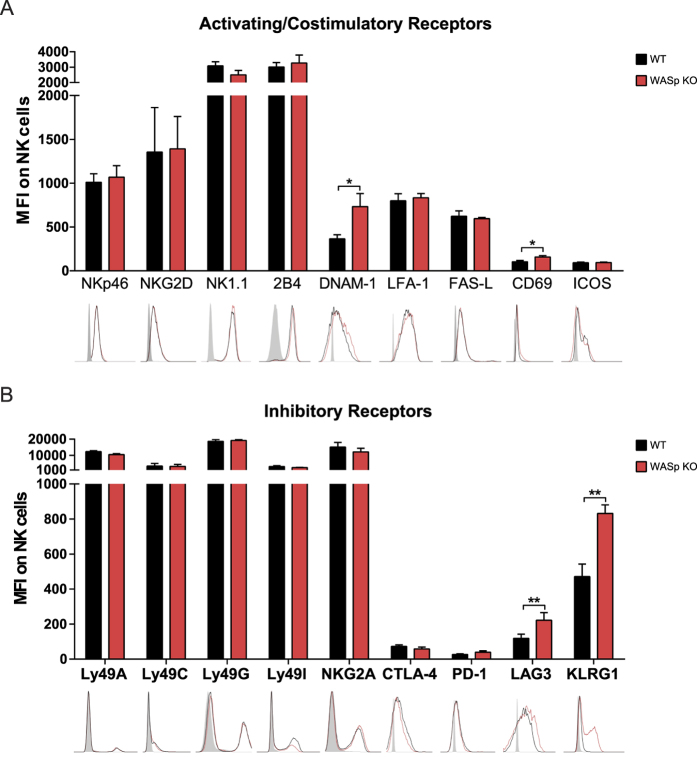
WASp KO NK cells have increased CD69, DNAM-1, LAG-3, and KLRG1. (**A**) Expression of activating and costimulatory receptors on C57Bl/6 WT and WASp KO NK1.1^+^CD3^−^ spleen NK cells. NKp46, NKG2D, NK1.1, 2B4, and DNAM-1; MFI is a pool of 3 individual experiments. WT n = 12, WASp KO n = 12. LFA-1, FAS-L, and ICOS; MFI is a pool of 2 individual experiments WT = 5, WASp KO = 5. CD69; MFI is a pool of 3 individual experiments. WT n = 10, WASp KO n = 11. The gray histogram represents an unlabeled control. (**B**) MFI of inhibitory receptors on C57Bl/6 WT and WASp KO NK1.1^+^CD3^−^ spleen NK cells. Ly49A, Ly49C, Ly49G, Ly49I, NKG2A; MFI is a pool of 2 individual experiments is shown. WT n = 5, WASp KO n = 6. CTLA-4, PD-1, and LAG-3; MFI is a pool of 2 individual experiments. WT n = 10, WASp KO n = 10. KLRG1; MFI is a pool of 3 individual experiments. WT n = 10, WASp KO n = 11. The gray histogram represents an unlabeled control. Graphs show mean values ± SEM. Significance was assessed with the Mann-Whitney test. *P < 05, **P < 01, ***P < 001 and ns  =  not significant. Abbreviations: MFI; Mean Fluorescence Intensity.

Since many of the NK cell inhibitory receptors interact with host MHC class I molecules we first examined MHC class I expression on spleen cells. Wildtype and WASp KO T cells, B cells, dendritic cells, and NK cells had similar expression of MHC class I molecules (supplementary Fig. 2). We next determined the expression of activating and inhibitory receptors on NK cells by assessing mean fluorescence intensity by flow cytometry. Wildtype and WASp KO NK cells had similar expression of activating receptors NKp46, NK1.1, NKG2D, 2B4, FAS-L, LFA-1, and ICOS (Fig. 3A), and of inhibitory receptors Ly49A, Ly49C, Ly49G2, Ly49I, NKG2A, CTLA-4, and PD-1 (Fig. 3B). WASp KO NK cells in the spleen had a slight but significant increase of the early activation receptor CD69 when compared to wildtype NK cells (Fig. 3A). WASp KO NK cells had higher expression of the activating receptor DNAM-1 (Fig. 3A) that associates with LFA-1 on the NK cell surface and potentiates activating signals^28^. When compared to wildtype NK cells, WASp KO NK cells also showed higher expression of the inhibitory receptors LAG-3 and KLRG1 (Fig. 3B), associated with terminally differentiated cells and cellular exhaustion^10^. KLRG1 is preferentially expressed on the most mature NK cells and the majority of KLRG1-expressing cells in WASp KO mice were mature CD27^−^CD11b^+^ NK cells (supplementary Fig. 3A). Since NK cell development depends on expression of the transcription factors T-box transcription factor (T-bet) and Eomesodermin (Eomes)^29^, we examined whether WASp KO NK cells had increased T-bet and Eomes expression as an indication of a terminally differentiated and/or exhausted phenotype and found no evidence for this (supplementary Fig. 3B,C). Another feature of exhausted CD8^+^ T cells is increased intracellular content of Granzyme B and perforin^30^. Naïve wildtype and WASp KO NK cells had similar content of granzyme B and perforin (Supplementary Fig. 3D,E). Together, this data shows that the receptor repertoire in WASp KO NK cells was skewed towards increased expression of DNAM-1, LAG-3, and KLRG1.

### WASp KO NK cells fail to accumulate F-actin in the lytic synapse

**Figure 4:**
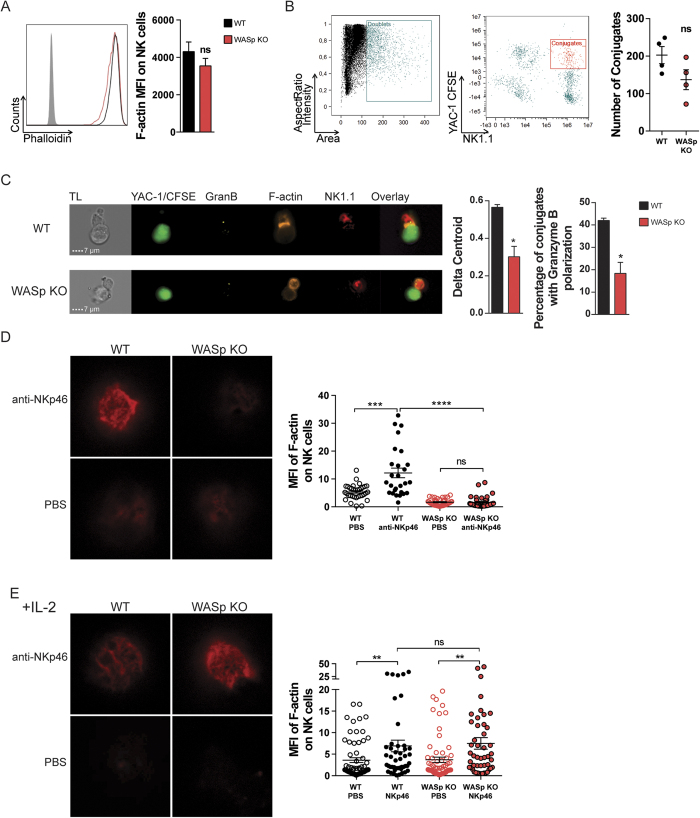
WASp KO NK cells fail to accumulate F-actin in the lytic synapse. (**A**) F-actin content in NK1.1^+^CD3^−^ NK cells in WT and WASp KO C57Bl/6 mice as assessed by intracellular phalloidin staining. The analysis represents a pool of 3 individual experiments (right). WT n = 9, WASp KO n = 8. The gray histogram represents an unlabeled control. (**B**) Gating strategy in imaging flow cytometry. Doublets were selected as a function of area and aspect ratio (*left*) and conjugates as a function of NK1.1 and CFSE (YAC-1) intensity (*right*). The absolute number of conjugates between WT or WASp KO NK cells and YAC-1 tumor cells, from a pool of 2 individual experiments. All conjugates were confirmed to consist of one NK cell and one YAC-1 cell by imaging analysis. WT n = 4, WASp KO n = 4. (**C**) *Left panel.* Lytic synapse formation between WT and WASp KO NK cells (NK1.1 in red) and YAC-1 tumor cells (CFSE in green). Granzyme B (yellow) and polymerized F-actin (orange) are shown. *Middle panel.* The delta centroid value indicates the extent of polarized fluorescence towards the synapse. *Right panel.* Percentage of NK cells with granzyme B located at the synapse out of total. A pool of 2 individual experiments is shown. WT n = 4, WASp KO n = 4. (**D**,**E**) F-actin polarization upon NKp46 activation (**D**) without IL-2 pre-treatment and (**E**) after IL-2 pre-treatment for 48 h. STED microscopy of the NK cell polarization of F-actin towards a surface coated with anti-NKp46 (top row) or PBS (bottom row). The graph shows quantification of F-actin MFI at the anti-NKp46-coated surface. The data represents a pool of 2 individual experiments. Dots indicate pictures analyzed in (**D**) WT PBS n = 34, WT NKp46 n = 27, WASp KO PBS n = 41, WASp KO NKp46 n = 39 from total WT n = 4, WASp KO n = 4 mice, and in (**E**) WT PBS n = 64, WT NKp46 n = 46, WASp KO PBS n = 65, WASp KO NKp46 n = 48) from total WT n = 4, WASp KO n = 4 mice. Graphs show mean values ± SEM. Significance was assessed with the Mann-Whitney test. *P < 05, ***P < 001, **** <0.0001, ns  =  not significant. Abbreviations: MFI; Mean Fluorescence Intensity.

Peripheral blood NK cells from WAS patient have decreased F-actin^31^. However, murine WASp-deficient NK cells had normal quantity of total F-actin as determined by flow cytometry (Fig. 4A). To visualize the lytic synapse, wildtype and WASp KO NK cells were co-incubated with YAC-1 lymphoma cells and analyzed by ImageStream analysis that combines flow cytometry with microscopy (Fig. 4B,C). The absolute number of conjugates between YAC-1 lymphoma cells and wildtype or WASp KO NK cells were similar as determined by flow cytometry (Fig. 4B) and microscopy (Fig. 4C). Lytic synapses between WT NK cells and YAC-1 cells were characterized by F-actin in the lytic synapse interface and polarized localization of granzyme B towards the lytic synapse (Fig. 4C). In contrast, WASp KO NK cells showed reduced capacity to accumulate F-actin and granzyme B towards the lytic synapse interface with YAC-1 tumor cells (Fig. 4C). To examine the capacity of NK cells to accumulate F-actin towards an interface surface in greater detail, wildtype and WASp KO NK cells were stimulated on glass surfaces coated with antibodies to the activating receptor NKp46 and imaged by stimulated emission depletion (STED) microscopy. Wildtype NK cells accumulated F-actin towards the interface surface (Fig. 4D), while WASp KO NK cells lacked F-actin towards the interface surface (Fig. 4D).

The cytolytic defect of WAS patient NK cells can be rescued by addition of exogenous IL-2^17^,^25^. Wildtype and WASp KO NK cells had similar expression of the IL-2 receptor complex and activation of NK cells with IL-2 rapidly induced phosphorylation of STAT5 (supplementary Fig. 4A,B). Moreover, addition of IL-2 to wildtype and WASp KO NK cells induced phosphorylation of WAVE2 (supplementary Fig. 4C). We examined if addition of IL-2 could restore the capacity to polarize actin towards activating receptors. WASp KO NK cells treated with IL-2 for 48 hrs showed normal polarization of polymerized actin towards anti-NKp46-coated glass surfaces (Fig. 4E). The lower capacity of WASp KO NK cells to organize the actin cytoskeleton and form lytic synapses could be completely rescued by stimulation with exogenous IL-2.

### IL-2 restores degranulation and IFNγ production by WASp KO NK cells

**Figure 5:**
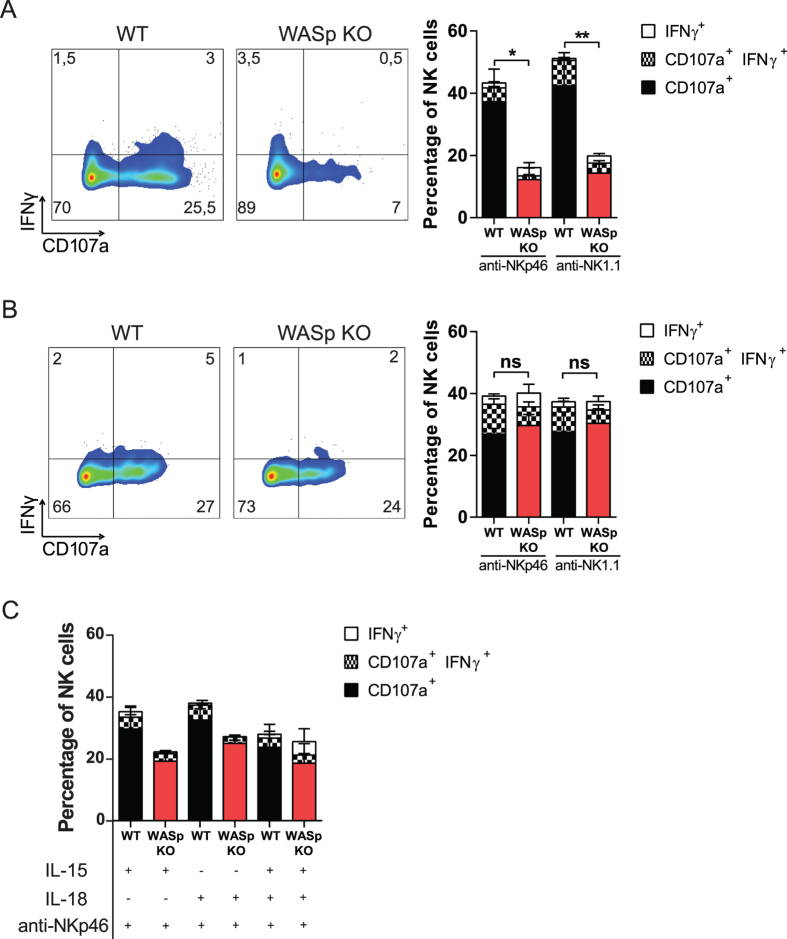
IL-2 restores degranulation and IFNγ production by WASp KO NK cells. (**A**) Degranulation and IFNγ production in C57Bl/6 NK1.1^+^CD3^−^ NK cells upon crosslinking the activating receptors NKp46 and NK1.1. Representative plots of the IFNγ production and degranulation (CD107a), in NK cells (*left*) and the analysis of a pool of 3 individual experiments (*right*). WT anti-NKp46 n = 9, WASp KO anti-NKp46 n = 5, WT anti-NK1.1 n = 5, WASp KO anti-NK1.1 n = 5. (**B**) NK cells were pretreated with IL-2 for 48 h and stimulated with anti-NKp46 and anti-NK1.1. The analysis of a pool of 3 individual experiments is shown. WT anti-NKp46 n = 8, WASp KO anti-NKp46 n = 9, WT anti-NK1.1 n = 5, WASp KO anti-NK1.1 n = 6. (**C**) NK cells were pretreated with IL-15, IL-18, or both for 48 h and thereafter stimulated with anti-NKp46. The analysis of a pool of 2 individual experiments is shown. WT n = 4, WASp KO n = 4. Graphs show mean values ± SEM. Significance was assessed with the Mann-Whitney test. *P < 05, **P < 01, ***P < 001, ns  =  not significant.

To investigate the functional outcome of decreased synapse formation by WASp KO NK cells, NK cells were stimulated with antibodies towards the activating receptors NKp46 and NK1.1, followed by measurement of IFNγ production and CD107a, as a marker for degranulation. Wildtype NK cells stimulated with anti-NKp46 or anti-NK1.1 antibodies degranulated, and produced IFNγ (Fig. 5A). In contrast, WASp KO NK cells had reduced proportion of CD107a^+^IFNg^+^ double positive cells upon NKp46 or NK1.1 stimulation (Fig. 5A). This was also associated with lower intracellular content of Granzyme B and perforin in WASp KO NK cells upon NKp46 activation (Supplementary Fig. 3D,E). We examined if the decreased degranulation and IFNγ response in WASp KO NK cells could be related to KLRG1 expression but found that KLRG1^−^, KLRG1^+^, and KLRG1^+^CD27^−^CD11b^+^ NK cells all showed a decreased response upon anti-NKp46 stimulation (supplementary Fig. 5). We next addressed if the impaired degranulation response of WASp KO NK cells could be rescued by addition of IL-2. Wildtype and WASp KO NK cells were pre-treated with IL-2 for 48 hours and then stimulated with anti-NKp46 or anti-NK1.1 antibodies to measure degranulation and IFNγ production. Pre-treatment with IL-2 before anti-NKp46 or anti-NK1.1 activation completely restored the degranulation capacity and IFNγ production by WASp KO NK cells (Fig. 5B). Moreover, pre-treatment of WASp KO NK cells with IL-15 and/or IL-18 also restored degranulation and IFNγ production to the response of wildtype NK cells (Fig. 5C), albeit not as efficiently as IL-2. Seeing that IL-2 restored the functionality of WASp KO NK cells, at least *in vitro*, we examined if IL-2 stimulation would influence expression of KLRG1. IL-2 pre-treatment for 48 hrs had no effect on KLRG1 expression by wildtype and WASp KO NK cells (supplementary Fig. 6A).

### IL-2 restores tumor rejection capacity by WASp KO NK cells

**Figure 6:**
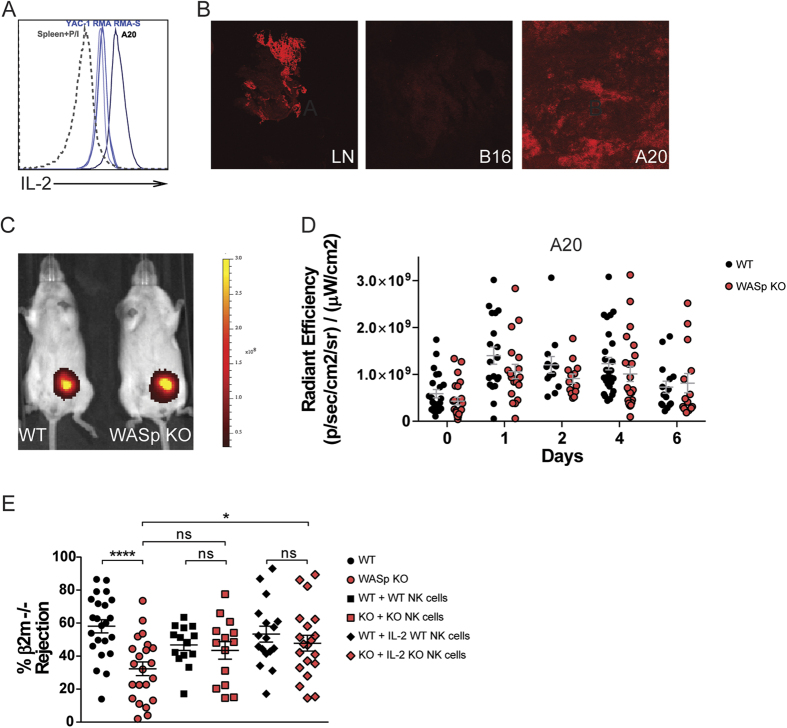
IL-2 restores tumor rejection capacity by WASp KO NK cells. (**A**) Intracellular staining for IL-2 in the A20, YAC-1, RMA and RMA-S tumor cells as assessed by flow cytometry. A representative histogram is shown and compared to splenocytes stimulated with PMA and Ionomycin (Spleen + P/I). (**B**) IL-2 staining on tissue sections at 10 ×  magnification. *Left*: Lymph node from a WT C57Bl/6 mouse. *Middle*: B16 melanoma from a WT C57Bl/6 mouse. *Right*: A20 lymphoma from a WT Balb/c mouse. (**C**) *In vivo* imaging of Balb/c mice injected with 1 × 10^6^ A20 lymphoma cells. Cells were labelled with the DiR dye to assess tumor growth, as a measure of fluorescence. Scale: Radiant efficiency in (p/sec/cm^2^/sr)/(mW/cm^2^). (**D**) A20 tumor growth in Balb/c mice plotted as radiant efficiency, from a pool of 4 individual experiments. Autofluorescence measured in a non-injected mouse was subtracted from injected mice at each time point. Day 0: WT n = 23, WASp KO n = 23, Day 1: WT n = 19, WASp KO n = 19, Day 2: WT n = 17, WASp KO n = 17, Day 4: WT n = 27, WASp KO n = 23, Day 6: WT n = 15, WASp KO n = 14. (**E**) NK cells were either *ex vivo* stimulated and expanded with IL-2 for 96 h or purified the day of injection without receiving any IL-2. C57Bl/6 mice that had received an injection of WT:β2m^−/−^ splenocytes 24 h earlier got either NK cells pre-treated with IL-2 or NK cells without IL-2 pre-treatment. *In vivo* splenocyte rejection is shown as percentage of rejected β2m^−/−^ splenocytes. The data is a pool of 6 individual experiments. Each dot represents one mouse. WT n = 23, WT + WT NK cells n = 14, WT + IL-2 WT NK cells n = 17, WASp KO n = 22, WASp KO + WASp KO NK cells = 14, WASp KO + IL-2 WASp KO NK cells = 20. Graphs show mean values ± SEM. Significance was assessed with the Mann-Whitney test. *P < 05, **P < 01, ***P < 001, ns  =  not significant.

We next examined if IL-2 production by lymphoma cells resulted in activation of WASp KO NK cells *in vivo*. When compared to spleen cells stimulated for 4 hours with PMA and Ionomycin (Spleen+P/I), all lymphoma cell lines used here (RMA, RMA-S, YAC-1 and A20 cells) produced high quantity of IL-2 without prior stimulation, with the A20 cells producing the highest quantity by intracellular staining and by measuring secretion of IL-2 (Fig. 6A and supplementary Fig. 6B). *In vivo*, A20 lymphoma tumors stained positively for IL-2 whereas B16 melanoma tumors were negative for IL-2 (Fig. 6B). To determine the response of WASp KO NK cells to lymphoma cells with high production of IL-2, we injected A20 lymphoma cells labeled with the DiR fluorescent dye into wildtype and WASp KO Balb/c mice and monitored tumor growth by IVIS during 6 days (Fig. 6C,D). WASp KO mice showed similar control of A20 tumor growth when compared to wildtype mice (Fig. 6C,D). We next tested if IL-2 pre-treatment *ex vivo* could restore functional capacity of WASp KO NK cells *in vivo*. Wildtype and WASp KO NK cells were stimulated and expanded with IL-2 for 96 hours *ex vivo* and then injected into mice that had received syngenic wildtype and MHC class I negative (β2m^−/−^) splenocytes, labeled with different concentrations of CFSE, 24 hours earlier. WASp KO NK cells stimulated *ex vivo* with IL-2 rejected β2m^−/−^ splenocytes similarly to wildtype NK cells (Fig. 6E). Transfer of WASp KO NK cells without prior IL-2 stimulation led to increased rejection capacity of β2m^−/−^ cells in WASp KO mice although the difference did not reach significance when compared to WASp KO mice that did not receive NK cells (Fig. 6E). This suggests that only increasing the number of WASp KO NK cells could rescue at least partly, the defect in rejection of b2m^−/−^ cells in WASp KO mice. Together, this data shows that IL-2 rescued dysfunction of WASp KO NK cells *in vivo*.

### WAS patient spleen NK cells have altered receptor expression

**Figure 7:**
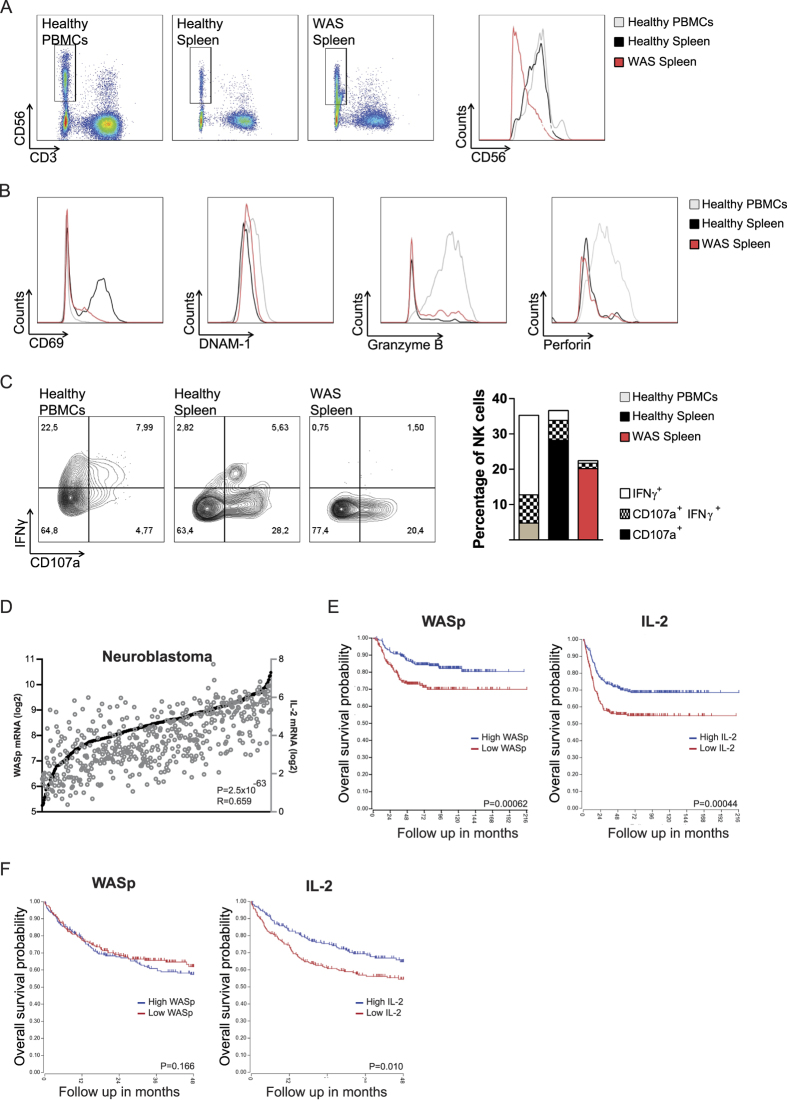
WAS patient spleen NK cells have altered receptor expression. (**A**) CD56 expression on healthy PBMCs, healthy splenocytes, and WAS patient splenocytes, plotted against CD3 in the plots and as MFI in the histograms. (**B**) CD69 and DNAM-1 surface expression, and Granzyme B and Perforin intracellular content in healthy PBMCs, healthy splenocytes, and WAS patient splenocytes. (**C**) Degranulation, as measured by CD107a surface expression and IFNγ production in healthy PBMCs, healthy splenocytes, and WAS patient splenocytes shown in contour plots (*left*) and a bar graph (*right*). (**D**–**F**) Expression analysis and survival of neuroblastoma patients in the R2 database, (R2: Genomics Analysis and Visualization Platform; http://r2.amc.nl). n = 498 (**D**) Correlation of WASp and IL-2 expression in neuroblastoma patients. Each patient is represented by circles for WASp expression (black circle; left Y-axis) and IL-2 expression (grey circle; right Y-axis). (**E**,**F**) Survival curves of (**E**) neuroblastoma patients and (**F**) diffuse large B cell lymphoma patients with high WASp expression (blue) and low WASp expression (red). Survival curves of (**E**) neuroblastoma patients and (**F**) diffuse large B cell lymphoma patients with high IL-2 expression (blue) and low IL-2 expression (red).

WAS patient NK cells isolated from peripheral blood mononuclear cells have decreased capacity to form the lytic synapse and kill target tumor cells *in vitro*^25^,^31^. To assess if these defects were also present in spleen, we examined spleen NK cells from a WAS patient and an age-matched healthy control. When compared to peripheral blood mononuclear cells with both CD56^bright^ and CD56^dim^ NK cells, spleen NK cells almost exclusively consisted of CD56^dim^ NK cells (Fig. 7A). When compared to healthy spleen NK cells, WAS patient NK cells had lower expression of CD56, leading to a large population of CD56^dim/negative^ NK cells (Fig. 7A). When compared to healthy spleen cells, WAS patient NK cells had lower expression of CD69, and increased expression of DNAM-1 and granzyme B (Fig. 7B). Perforin expression was similar in WAS patient and control spleen NK cells (Fig. 7B). KLRG1 was not detected on spleen NK cells. WAS patient spleen NK cells had decreased degranulation response as indicated by lower proportion of CD107a^+^IFNγ^+^ NK cells upon PMA+Ionomycin stimulation and when compared to healthy donor spleen NK cells (Fig. 7C). Since we were not able to obtain tumor tissue from WAS patients, we next wanted to examine if we could correlate expression of WASp and IL-2 with tumor outcome in cancer patients. NK cells can kill neuroblastoma cells in a process dependent on DNAM-1^32^. Since both WASp KO NK cells and WAS patient NK cells had increased expression of DNAM-1, we examined correlation between expression of WASp and IL-2 with survival in a cohort of neuroblastoma patients in the R2 database (R2: Genomics Analysis and Visualization Platform; http://r2.amc.nl). By analysing a non-hematopoietic derived tumor, neuroblastoma, we focused on gene expression of WASp and IL-2 by tumor-infiltrating hematopoietic cells. In this cohort, expression of WASp correlated with expression of IL-2 (Fig. 7D). When comparing neuroblastoma patients with high expression of WASp with those that had low or absent expression of WASp, high expression of WASp correlated with better survival (Fig. 7E). Using the same analysis, high expression of IL-2 correlated with better survival in the neuroblastoma patient cohort (Fig. 7E). We next analysed a cohort of patients with diffuse large B cell lymphoma using the R2 database. Since diffuse large B cell lymphoma is of hematopoietic cell origin and expresses WASp, it was not surprising that high expression of WASp if anything led to increased mortality (Fig. 7E). Interestingly, high expression of IL-2 correlated with better survival also in the cohort of diffuse large B cell lymphoma patients (Fig. 7F).

## Discussion

Current data about the function of WASp-deficient NK cells during tumorigenesis is somewhat contradictory. Using *in vivo* studies of WASp KO mice, Catucci *et al.* has shown that WASp KO mice have increased metastasis of B16 melanoma tumor cells^23^, whereas Ishihara *et al.* detected decreased metastasis of breast carcinoma cells in WASp KO mice^24^. Catucci *et al.* shows that defective NK cell killing could be a secondary cause of decreased cross-talk between WASp KO dendritic cells and NK cells, required for the priming of NK cells and polarized cytokine release^23^,^33^. The study by Ishihara *et al.* show that tumor-associated macrophages from WASp KO mice showed reduced migration resulting in decreased tumor metastasis^24^. The previous studies describe tumorigenesis in WASp KO mice to non-hematopoietic derived tumors, B16 melanoma and breast cancer cells^23^,^24^. Since almost 90% of the malignancies in WAS patients are of lymphoreticular origin, we took the approach to study the killing response by WASp KO NK cells to lymphoma cells and hematopoietic grafts. We found that WASp KO NK cells had decreased capacity to kill MHC class I negative hematopoietic grafts. In light of this finding and the marked defects in the *in vitro* capacity to degranulate and produce IFNγ, it was surprising that WASp KO NK cells could control growth of lymphoma cells such as A20, YAC-1, and RMA-S cells *in vivo*. We reason that this could be due to that tumor cells of lymphoid origin express multiple activating ligands and/or express high quantity of cytokines such as IL-2 that rescue dysfunction of WASp KO NK cells (this study)^17^,^25^. This may explain why WAS patients have relatively late onset of tumor development in adolescence and adulthood^18^,^19^,^20^,^21^,^22^,^34^.

WASp KO NK cells showed normal distribution of the inhibitory receptor repertoire but had decreased capacity to reject MHC class I negative grafts. This suggests that despite having normal education in terms of inhibitory receptor repertoire, loss of MHC class I molecules was not enough to trigger activation of WASp KO NK cells in the wildtype:β2m^−/−^ splenocyte rejection assay. The exact details on where NK cells are educated and which host cells they interact with remains enigmatic. It has been suggested that the localization of activating and inhibitory receptors to specific membrane microdomains steer education so that when activating and inhibitory receptors cluster together NK cells become uneducated and hypo-responsive^35^. If instead clustering of activating and inhibitory receptors occurs in separate membrane microdomains, NK cells become educated and responsive^35^,^36^. Our data suggests that the synapses formed during education and effector stages are regulated by different signaling molecules and that the education synapse occurs independently of WASp while the lytic synapse requires WASp for optimal signaling. This is similar to the requirement for WASp during development and function of B and T cells, where immune synapse formation is critical both during development and in cell-to-cell communication in peripheral organs. During development of B and T cells, WASp is largely dispensable and another WASp family member, neuronal(N)-WASp, acts redundantly with WASp^37^,^38^. It is possible that N-WASp serves redundant function with WASp or has a unique role in development and education of NK cells.

CD69 is an early activation receptor upregulated on NK cells and T cells in the early immune response. Murine WASp KO NK cells showed higher expression of CD69, while WAS patient spleen NK cells instead showed lower expression of CD69. In the murine setting, we examined CD69 on NK cells from naïve mice. In contrast, WAS patient spleen cells were probably subjected to a state of chronic inflammation as evidenced by a large proportion of WAS patient NK cells having low or absent expression of CD56 when compared to CD56^dim^ NK cells in spleen cells from the healthy control. CD56^negative^ NK cells is a rare population in healthy individuals but are expanded in chronically infected individuals^39^. When compared to CD56^dim^ NK cells, CD56^negative^ NK has lower capacity to degranulate and produce cytokines such as IFNγ^39^. We show here that WAS patient spleen NK cells showed decreased capacity to degranulate and produce IFNγ in response to PMA and Ionomycin. The limited WAS patient spleen sample did not allow us to examine receptor-mediated degranulation and IFNγ production. Murine WASp KO NK cells degranulated and produced IFNγ upon PMA and Ionomycin activation. Together with the mild defect in WAS patient spleen NK cell in response to PMA and Ionomycin, this suggest that WASp is required for proximal receptor signaling leading to degranulation and IFNγ production.

Of all the activating receptors tested in our panels, WASp KO NK cells and WAS patient NK cells had increased expression of DNAM-1. DNAM-1 is a key molecule in differentiation of memory NK cells and blocking DNAM-1 suppresses NK cell memory formation during cytomegalovirus infection^40^. Interestingly, when comparing DNAM-1^+^ with DNAM-1^−^ NK cells and their tumor rejection capacity and *in vitro* killing, DNAM-1^+^ NK cells showed superior rejection and tumor killing capacity of B16 melanoma cells and RMA-S lymphoma cells^41^,^42^. It is possible that increased DNAM-1 expression on WASp KO and WAS patient NK cells leads to better killing of tumor cells that express DNAM-1 ligands, which are members of the Nectin/Nectin like family of adhesion molecules^43^, such as B16 melanoma cells and RMA-S lymphoma cells. Of all the inhibitory receptors tested here, only KLRG1 and LAG-3 were markedly increased on WASp KO NK cells. Expression of KLRG1 and LAG-3 have been associated with an exhausted phenotype in CD8^+^ T cells and in NK cells^10^,^44^, implying that WASp KO NK cells may show signs of exhaustion. However, WASp KO NK cells had capacity to reject A20 and RMA-S lymphoma cells, and controlled growth of YAC-1 cells. Moreover, WASp KO NK cells remained responsive to IL-2 stimulation as determined by STAT5 signaling, WAVE2 activation, and rescued ability to polarize actin, degranulate, and produce IFNγ in response to IL-2. WASp KO NK cells showed normal expression of the transcription factors T-bet and Eomes and had similar content of granzyme B and perforin, suggesting that they did not have an exhausted phenotype^29^,^30^. Interestingly, a recent study shows that terminally matured CD27^low^KLRG1^+^ NK cells are needed for effective anti-tumor immunity^14^. WASp KO NK cells had high expression of KLRG1 in mature CD27^low^CD11b^+^ NK cells when compared to wildtype mice. Together, our data suggests that WASp KO NK cells were not exhausted but that they need increased signaling to reach activation threshold and that loss of MHC class I alone was insufficient to trigger a killing response by WASp KO NK cells. The analysis of WASp KO NK cells implies that increased expression of KLRG1, LAG-3 and DNAM-1 was associated with normal tumor rejection capacity, at least when multiple ligands and cytokines such as IL-2 was produced by tumor cells.

Several strategies have been tested to target NK cells in cancer therapy^3^. All strategies aim at skewing the signaling balance towards activating and away from inhibitory input using cytokine treatment and/or transfer of *ex vivo* expanded NK cells^3^,^45^,^46^. We provide evidence that both these strategies may be viable to rescue NK cell dysfunction in WAS patients. IL-2 treatment of WASp KO NK cells completely restored degranulation capacity and IFNγ production *in vitro*, and IL-2 treatment of WASp KO NK cells *ex vivo* stimulated killing capacity of MHC class I negative hematopoietic grafts *in vivo*. Our data is promising in the light of that the responsiveness of NK cells remains plastic once the cells reach maturity and can be reset when the NK cells are exposed to an environment in which MHC class I expression differs^47^. NK cells are sensitive to the balance of the inhibitory and activating ligands, but once activation is reached, killing is an all-or-nothing response^15^,^48^. Targeted cytokine therapy may be an option to restore cellular dysfunctions in WAS as has been shown for many primary immunodeficiency diseases^49^. We show here that IL-2, IL-15 and IL-18, that signal via the common γ chain, restores functionality of murine WASp KO NK cells. Subcutaneous administration of IL-2 is currently in clinical trial for WAS patients and has shown positive outcome in one WAS patient^17^. To avoid activation of regulatory T cells via IL-2 binding to the high affinity IL-2 receptor α chain, a modified version of IL-2 capable of functioning independently of the α chain has been used successfully to activate NK cell killing of MHC class I negative tumors^45^,^46^,^50^. A more feasible option may be *ex vivo* culture of NK cells in the presence of IL-2 where the gain is two-fold, rescue of NK cell dysfunction, such as in WASp deficiency, and expansion of NK cells^3^,^51^.

## Methods

### WAS patient cells

Splenocytes were obtained from a previously defined patient with WAS due to complete deletion of exons 1–12 of WASp^52^ or from a normal person undergoing splenectomy due to trauma. Control PBMCs were isolated from a healthy volunteer. Blood and the tissue were collected under protocols including written informed consent and approved by the Institutional Review Boards of the Immune Disease Institute (formerly the Center for Blood Research; Boston, MA, USA). PBMCs and splenocytes were used from cryopreserved samples where indicated. For flow cytometry, single-cell suspensions were labeled with fluorescently conjugated anti–human antibodies: CD56 (318334, Biolegend), CD3 (317328, Biolegend), DNAM-1 (338304, Biolegend), CD69 (562617, BD Biosciences), Perforin (556577, BD Biosciences), CD107a (15–107942, eBioscience), Granzyme (GRB17, Invitrogen), and IFNγ (502520, Biolegend). Expression data in neuroblastoma diffuse large B cell lymphoma patients was acquired from the R2 database (R2: Genomics Analysis and Visualization Platform; http://r2.amc.nl).

### Mice

WASp KO mice on C57Bl/6 (H-2^b^) and Balb/c (H-2^d^) background were backcrossed for at least 9 generations. Balb/c mice were used for the *in vivo* imaging experiments and all remaining experiments were performed with C57Bl/6 mice. WASp KO mice, littermate wildtype controls, and β2 microglobulin^−/−^ (β2m^−/−^; H-2^b^) mice were bred and maintained at the animal facility of the Department of Microbiology, Tumor and Cell Biology at Karolinska Institutet under specific pathogen-free conditions. Mice were used 6–12 weeks of age and all animal experiments were performed after approval from the local ethical committee (the north Stockholm district court) and performed in accordance with national and institutional guidelines.

### Antibodies, Flow Cytometric analysis, and Immunohistochemistry

For flow cytometry, single-cell suspensions were labeled with fluorescently conjugated anti–mouse antibodies: CD3 (145.2C11), CD27 (29A1.4), CD11b (M1/70.15), CD69 (H1.2F3), CD19 (6D5), Ly6D (49-H4), CD244 (m2B4(B6)458.1), cKit (2B8), CD127 (A7R34), CD122 (TM-β1), CD135 (A2F10), Ly49A (YEI/48), CD25 (PC61), CD11a (H155-78), DNAM1 (TX42.1), PD-1. ICOS (7E.17G9), CTLA-4 (UC10-4B9), LAG3 (C9B7W), and CD132 (TUGm2) (all Biolegend), NK1.1 (PK136), KLRG1 (2F1), CD107a (1D4B), Ly49G2 (4D11), and NKG2D (CX5) (all BD Biosciences), NKp46 (29A1.4) (Life Technologies) and Ly49I (YLI-90) and NKG2A (20d5) (eBioscience). The 4LO3311 (Ly49C) hybridoma was a kind gift from Suzanne Lemieux. All surface staining was performed in phosphate-buffered saline (PBS) after blocking unspecific staining via FcγRII/III with purified anti-CD16/32 (2.4G2). Dead cells were excluded from the gating with the Aqua dead cell dye (Life Technologies). For intracellular staining of IL-2 and IFNγ, cells were fixed and permeabilized with the Cytofix/Cytoperm kit (BD) and then stained with IL-2 (JES6-5H4, Biolegend) and IFNγ (XMG1.2, BD Biosciences) antibodies. For pWAVE2 and pSTAT5 analysis, cells were stimulated with IL-2 for the indicated time, fixed with Lyse/Fix (BD Biosciences), permeabilized with Perm Buffer III (BD Biosciences) and stained with pWAVE2 (WP1821 Tyr-150, ECM Biosciences) and pSTAT5 (47/Stat5(pY694), BD Biosciences) antibodies. Secretion of IL-2 was determined during 45 minutes using the IL-2 secretion assay detection kit (Miltenyi). All flow cytometric analyses were performed on an LSRII or LSRFortessa X-20 (Becton Dickinson) and data analysis was performed using the FlowJo software (Tree Star). For immunohistochemistry, tissue sections were stained with IL-2-biotin (JES6-5H4, Biolegend) and Streptavidin (Life Technologies) antibodies. Images were collected with a Leica DM IRBE confocal laser scanning microscope (Leica Microsystems) equipped with 1 argon and 2 HeNe lasers, using an HC PL APO lens at 10x/0.40 CS and 90% glycerol (MP Biomedicals) and processed with Adobe Photoshop CS5 (Adobe Systems) and ImageJ software (National Institutes of Health).

### Tumor cell injection and *In vivo* imaging

A20 B cell lymphoma cells or YAC-1 T cell lymphoma cells were labelled with the DiR dye (Life Technologies) and 1 × 10^6^ cells were injected subcutaneously or intravenously, respectively, into Balb/c WT and WASp KO mice. *In vivo* imaging was performed immediately after injection, and at Day 1, 2, 4 and 6 for the A20 experiments and after 5 h for the YAC-1 experiments. *In vivo* imaging of fluorescence was performed with a CCD camera, mounted in a light-tight chamber (IVIS Spectrum CT, Caliper LifeSciences). Anesthesia was induced by 4% isofluorane and maintained by 2.3% isofluorane throughout the imaging procedure. Regions of interest were localized around the injections sites and intensity was quantified as Radiant Efficiency in (p/sec/cm^2^/sr)/(mW/cm^2^). Analysis was performed using the Living Image^®^ software version 4.1 (Caliper Life Sciences). For immunohistochemistry of A20 and B16 melanoma cells, 1 × 10^6^ tumor cells were injected subcutaneously in Matrigel (BD Biosciences) and removed on day 7 for analysis of IL-2 expression.

### *In vivo* rejection assay

Spleens from WT and β2m^−/−^ mice were mechanically disrupted, erythrocyte depleted, and the single cell suspensions were labelled with a high (β2m^−/−^) or low (WT) dose of CFSE at a 1:1 ratio^48^,^53^. RMA and RMA-S (a TAP-2 deficient variant of RMA with low expression of MHC class I molecules) T cell lymphoma cells were grown as ascites in irradiated C57Bl/6 mice and collected to be labelled with a high (RMA-S) or low (RMA) dose of CFSE. The cells were mixed in a 1:1 ratio and injected intravenously at 30 × 10^6^ cells into WT or WASp KO C57Bl/6 mice. After 2 days, spleens from these mice were collected and the amount of rejected β2m^−/−^ or RMA-S cells was assessed by flow cytometry and calculated as follows: 100% − (β2m^−/−^ or RMA-S cells in sample/WT or RMA cells in sample)/(β2m^−/−^ cells or RMA-S in inoculate/WT or RMA cells in inoculate).

### *In vitro* NK cell stimulation

Splenocytes from WT and WASp KO C57Bl/6 mice were mechanically disrupted, erythrocyte depleted, and the cells were added to a plate pre-coated with 20 μg/ml anti-NKp46 antibody (R&D Systems), or 50 μg/ml anti-NK1.1 antibody (BD Biosciences) for 4 h at 37 °C. Cells were incubated in complete RPMI medium, supplemented with 10 mg/ml Brefeldin A (GolgiPlug; BD Biosciences) and anti-CD107a. Positive controls were stimulated with 20 ng/ml PMA (Sigma) and 50 ng/ml Ionomycin (Sigma). Cells were then harvested and stained for surface markers and intracellular IFNγ after fixation and permeabilization with the Cytofix/Cytoperm kit (BD Biosciences) and analyzed by flow cytometry. Degranulation was assessed by the surface expression of CD107a.

### IL-2 treatment

Splenocytes from WT and WASp KO C57Bl/6 mice were obtained as described above and cultured in complete RPMI, at 2 × 10^6^ cells/ml with 1000 U/ml IL-2 (Peprotech) for 48 h (*in vitro* experiments) or 96 h (*in vivo* experiments).

### Synapse Formation and Imaging Flow Cytometry

Splenic NK cells from WT or WASp KO C57Bl/6 mice were purified by MACS sorting with CD49b beads (Miltenyi Biotech). NK cells were incubated with YAC-1 lymphoma cells at a 5:1 effector:target ratio for 1 h at 37 °C. YAC-1 cells were pre-labelled with CFSE and NK cells with anti-NK1.1. anti-CD3 and the Aqua dead cell dye were used to exclude contaminating T cells and dead cells. YAC-1 - NK cell conjugates were fixed and permeablized (Cytofix/Cytoperm kit; BD Biosciences) and stained with Phalloidin (Life Technologies) and anti-Granzyme B (GB12, Invitrogen). The conjugates were acquired on an ImageStream^X^ Mark II (Amnis) and gated as NK1.1^+^CFSE^+^ doublets. Analysis was performed using the IDEAS v.6 software (Amnis).

### Stimulated Emission Depletion (STED) Microscopy

NK cells from WT and WASp KO C57Bl/6 mice were stimulated for 4 h on glass coverslips, coated with 20 μg/mL anti-NKp46 antibodies or PBS. The cells were then fixed, permeabilized, and labeled with Phalloidin-Alexa Fluor 488 to detect F-actin and NK1.1-APC. The imaging was performed on a Leica TCS SP8 microscope with a time-gated stimulated emission depletion (STED) module. Image analysis was performed on the Image J software.

### Statistics

All statistical analyses were performed using the Prism software (version 6, GraphPad). Results are expressed as means ± SEM. All data was analyzed by ROUT (Q = 1.0%) and outliers were excluded as indicated in the Fig. legends. Differences between individual groups were analyzed for statistical significance using the non-parametrical Mann-Whitney test. *P < 0.05; **P < 0.01; ***P < 0.001; ns, not significant.

## Additional Information

**How to cite this article**: Kritikou, J. S. *et al.* IL-2 in the tumor microenvironment is necessary for Wiskott-Aldrich syndrome protein deficient NK cells to respond to tumors *in vivo.* *Sci. Rep.* **6**, 30636; doi: 10.1038/srep30636 (2016).

## Supplementary Material

Supplementary Information
