# Supplementary material for: IL-2 in the tumor microenvironment is necessary for Wiskott-Aldrich syndrome protein deficient NK cells to respond to tumors in vivo
Source: Sci Rep. 2016 Aug 1;6:30636. doi: 10.1038/srep30636 (PMC4967920; doi:10.1038/srep30636)
Supplement: Supplementary Information [file srep30636-s1.doc]

**IL-2 in the tumor microenvironment is necessary for Wiskott-Aldrich syndrome protein deficient NK cells to respond to tumors *in vivo***

**Joanna S. Kritikou^*^, Carin I.M. Dahlberg^*^, Marisa A.P. Baptista ^*^, Arnika K. Wagner ^*^, Pinaki P. Banerjee^†^, Lavesh Amar Gwalani^†^, Cecilia Poli^†^, Sudeepta K. Panda^¤^, Klas Kärre^*^, Susan M. Kaech^‡,§^, Fredrik Wermeling^¤^, John Andersson^¤^, Jordan S. Orange^†^, Hanna Brauner^*^, Lisa S. Westerberg^*^**

^*^Department of Microbiology Tumor and Cell biology, Karolinska Institutet, Stockholm 171 77, Sweden; ^†^Center for Human Immunobiology, Baylor College of Medicine and Texas Children's Hospital, Houston, TX 77030, USA; ^‡^Department of Immunobiology, Yale University School of Medicine, New Haven, CT 06520, USA; ^§^Howard Hughes Medical Institute, 4000 Jones Bridge Road, Chevy Chase, MD 20815, USA; ^¤^Department of Medicine Solna, Karolinska Institutet, and Karolinska University Hospital, Stockholm 171 76, Sweden

**
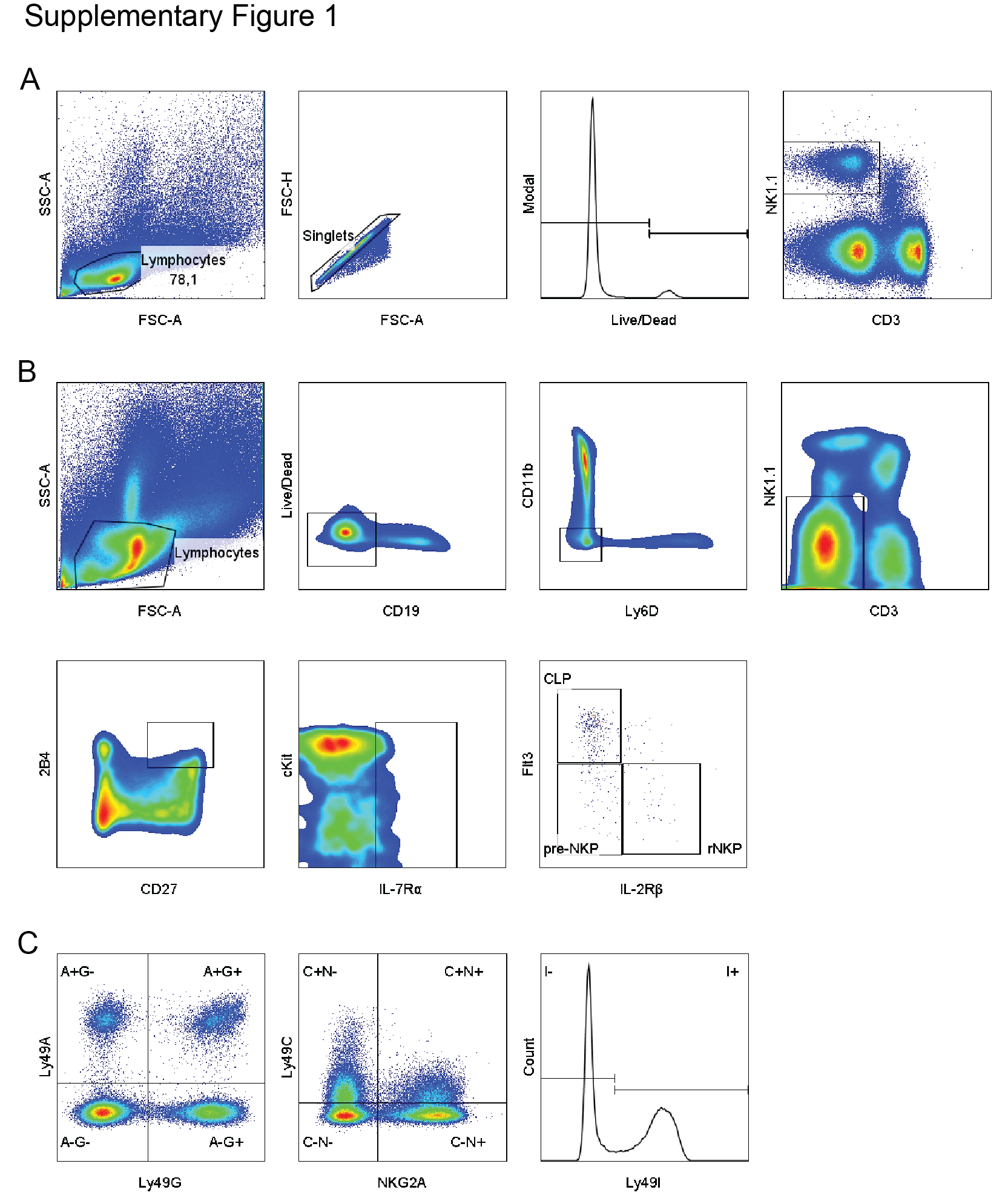
**

**Supplementary Fig. 1.** **Gating strategies. (A)** The gating strategy used for NK cells. NK cells are defined as Lymphocytes/Single cells/Alive cells/NK1.1^+^CD3^-^ throughout this article. **(B)** The gating strategy for bone marrow development in Figure 2A. CLP, Pre-NKP and NKP cells are defined as Lymphocyte/Alive/CD19^-^CD11b^-^Ly6D^-^NK1.1^-^CD3^-^c-Kit^-^2B4^+^CD27^+^IL-7Rα^+^ bone marrow cells and then based on Flt3 and IL-2Rβ expression. CLPs are Flt3^+^IL-2Rβ^-^, Pre-NKPs are Flt3^-^ IL-2Rβ^-^, and NKPs are Flt3^-^ IL-2Rβ^+^. **(C)** The gating strategy used for the inhibitory receptor analysis on NK cells. Ly49A and Ly49G expression is defined in the first plot and Ly49C and NKG2A in the second plot, which is further defined as Ly49I positive or negative in the third plot.


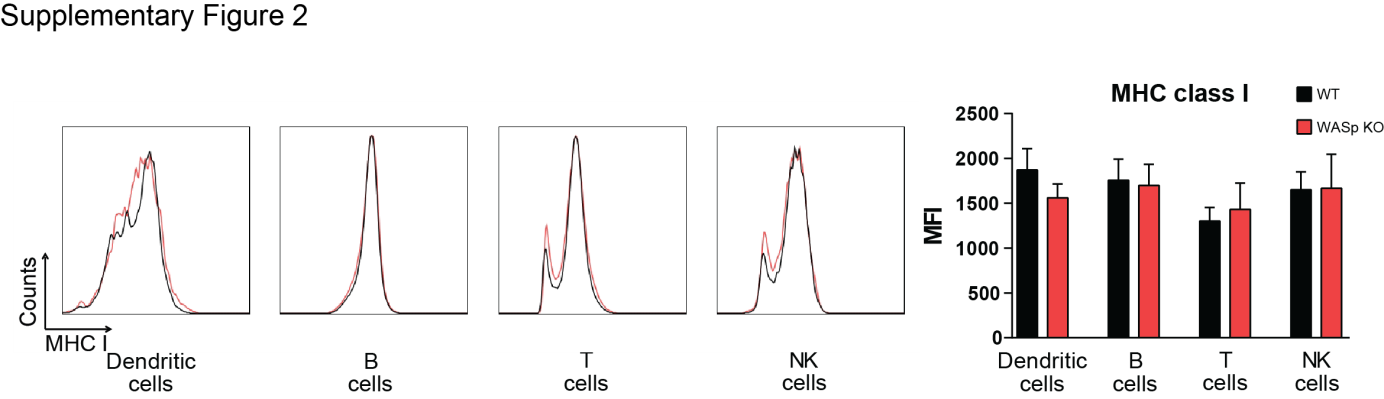


**Supplementary Fig. 2.** MHC class I expression on splenic CD11c^+^ dendritic cells, B220^+^ B cells, CD3^+^ T cells, and NK1.1^+^CD3^-^ NK cells from C57Bl/6 WT and WASp KO mice. The data is a pool of 2 individual experiments. Each dot represents one mouse. WT n=6, WASp KO n=6. Graphs show mean values±SEM. Significance was assessed with the Mann-Whitney test and showed no significant difference found in MHC Class I expression. Abbreviations: MFI; Mean Fluorescence Intensity.


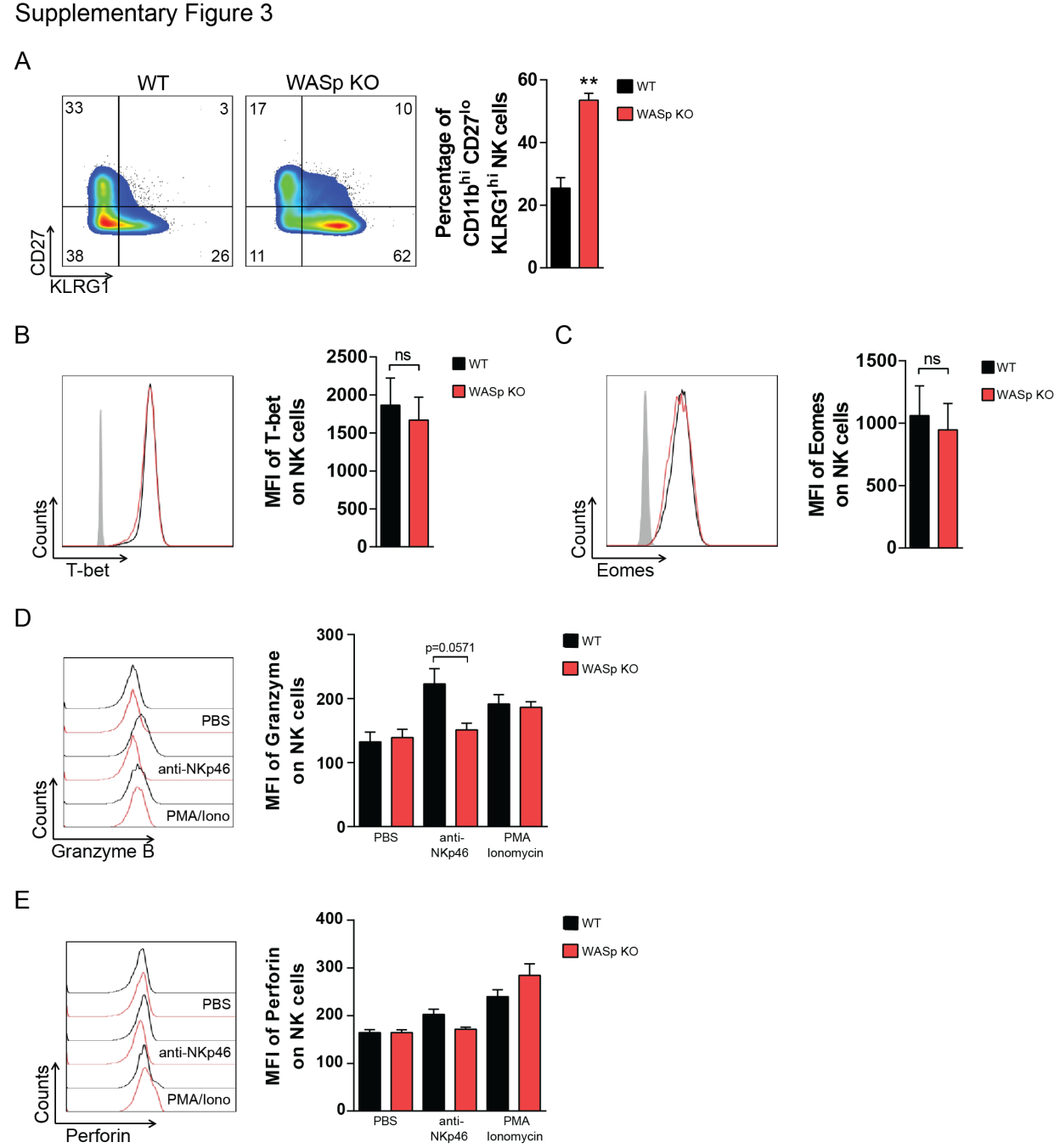


**Supplementary Fig. 3. Expression of KLRG1, T-bet, Eomes, Granzyme B, and Perforin. (A)** KLRG1 expression on mature C57Bl/6 NK cells. NK1.1^+^CD3^-^CD11b^+^ NK cells were gated on CD27 and KLRG1. The analysis of a pool of 2 individual experiments is shown. WT n=6, WASp KO n=7. **(B)** T-bet expression in C57B/6 WT and WASp KO NK1.1^+^CD3^-^  NK cells. Representative histograms of T-bet in NK cells (left) and the analysis of a pool of 2 individual experiments (right). WT n=6, WASp KO n=6. The gray histogram represents an unlabeled control. **(C)** Eomes expression in C57B/6 WT and WASp KO NK1.1^+^CD3^-^ NK cells. Representative histograms of Eomes in NK cells (left) and the analysis of a pool of 2 individual experiments (right). WT n=6, WASp KO n=6. The gray histogram represents an unlabeled control. **(D)** Granzyme B intracellular content in naïve C57Bl/6 WT and WASp KO NK1.1^+^CD3^-^ NK cells. Representative histograms of Granzyme B in naïve, anti-NKp46 stimulated and PMA/Ionomycin stimulated NK cells (*left*) and the analysis of a pool of 2 individual experiments (*right*). WT n=6, WASp KO n=6. **(E)** Perforin intracellular content in naïve and stimulated C57Bl/6 WT and WASp NK1.1^+^CD3^-^ KO NK cells. Representative histograms of Perforin in naïve, anti-NKp46 stimulated and PMA/Ionomycin stimulated NK cells (*left*) and the analysis of a pool of 2 individual experiments (*right)*. WT n=6, WASp KO n=6. Graphs show mean values±SEM. Significance was assessed with the Mann-Whitney test. ns = not significant. Abbreviations: MFI; Mean Fluorescence Intensity.


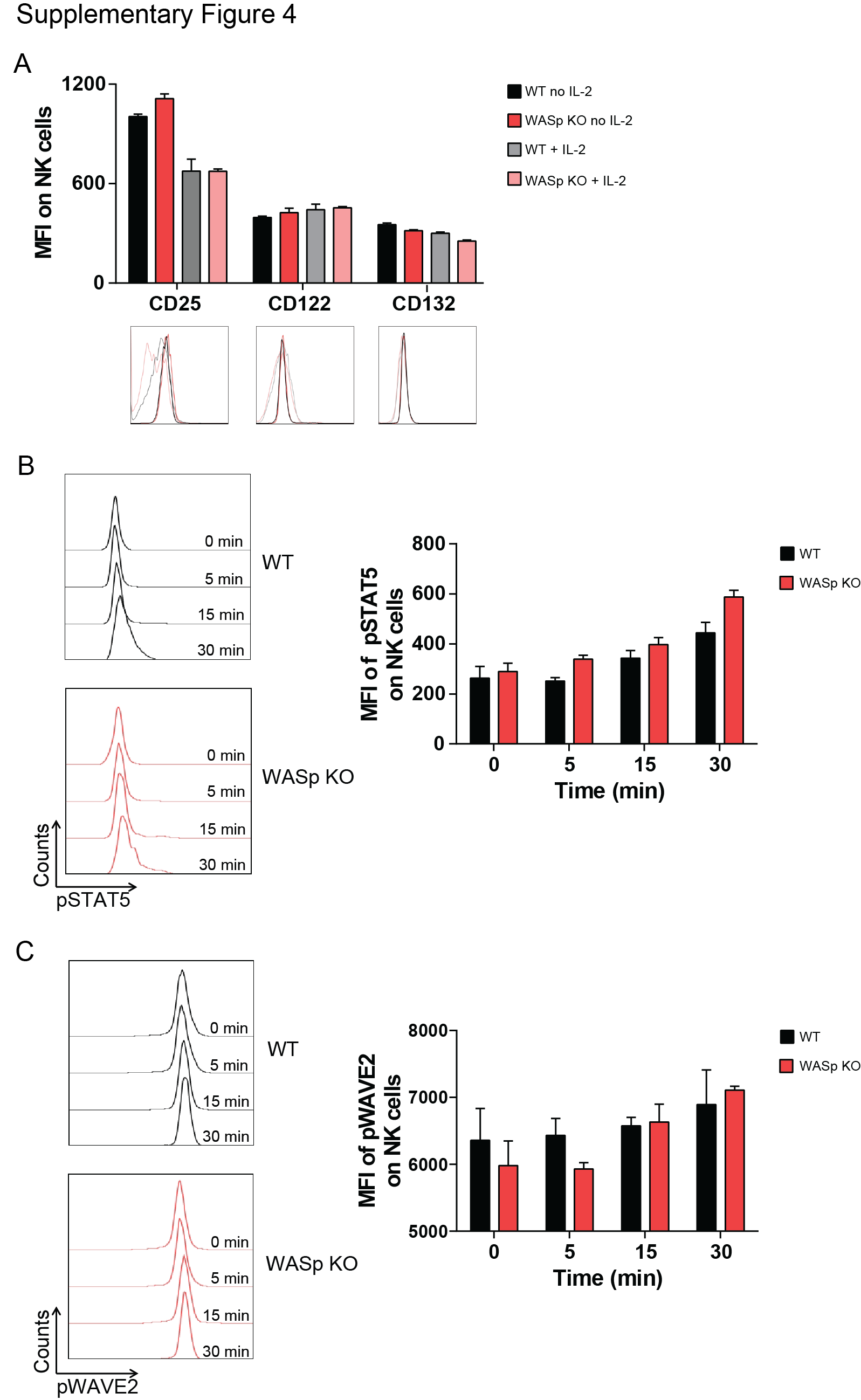


**Supplementary Fig. 4. IL-2R complex, and STAT5 and WAVE2 phosphorylation before and after IL-2 treatment. (A)** IL-2Rα (CD25) expression is lower after 48h IL-2 treatment in both WT and WASp KO C57Bl/6 NK1.1^+^CD3^-^ NK cells but IL-2Rβ (CD122), and IL-2Rγ (CD132) stay the same. WT n=6, WASp KO n=6. **(B)** STAT5 phosphorylation (pSTAT5) is induced after IL-2 treatment in both WT and WASp KO C57Bl/6 NK cells. WT n=4, WASp KO n=3. **(C)** WAVE2 phosphorylation (pWAVE2) is induced after IL-2 treatment in both WT and WASp KO C57Bl/6 NK cells. WT n=3, WASp KO n=4. Graphs show mean values±SEM. Abbreviations: MFI; Mean Fluorescence Intensity.


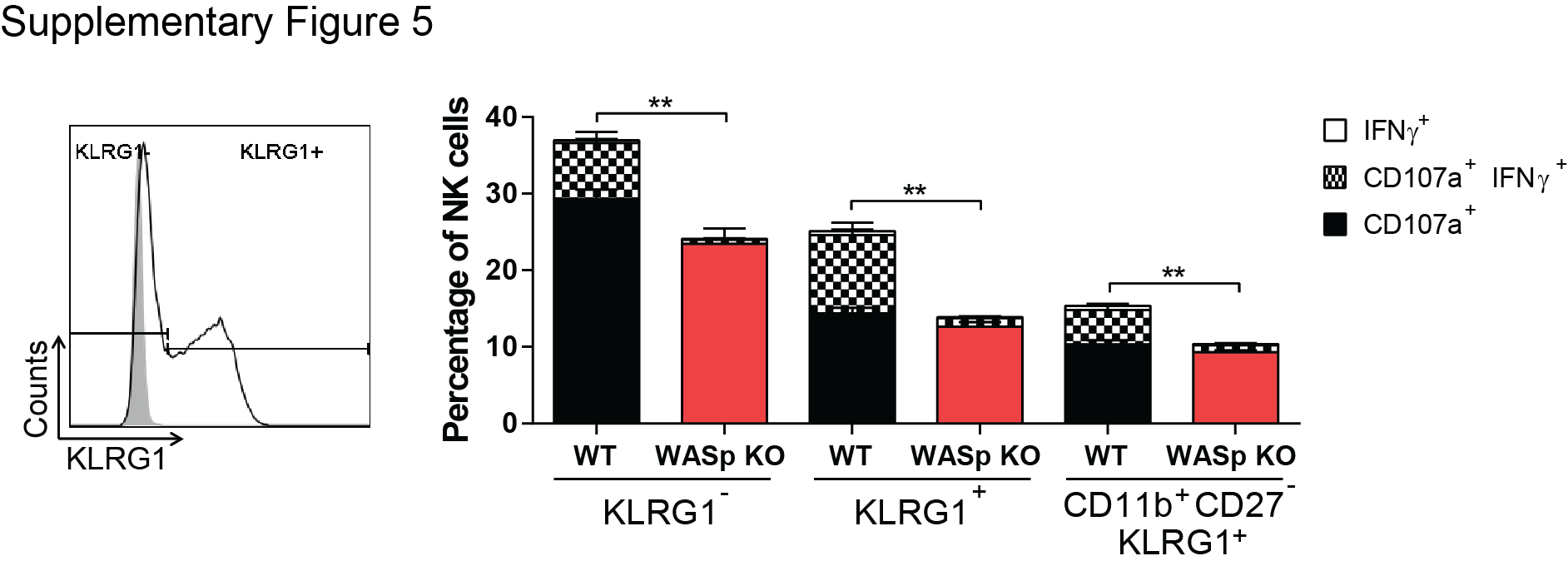


**Supplementary Fig. 5. KLRG1 expression and response to anti-NKp46 stimulation.** The gating strategy for KLRG1 positive and negative NK1.1^+^CD3^-^ NK cells. The gray histogram represents the FMO control. Degranulation (CD107a) and IFNγ production upon stimulation by crosslinking the activating receptor NKp46 in 3 different NK1.1^+^CD3^-^ NK cell populations; KLRG1^-^, KLRG1^+^, and KLRG1^+^CD11b^+^CD27^-^ NK cells. WT n=5, WASp KO n=5. Graphs show mean values±SEM. Significance was assessed with Mann-Whitney test. ** P < 0.01. Abbreviations: MFI; Mean Fluorescence Intensity. FMO; Fluorescence Minus One.


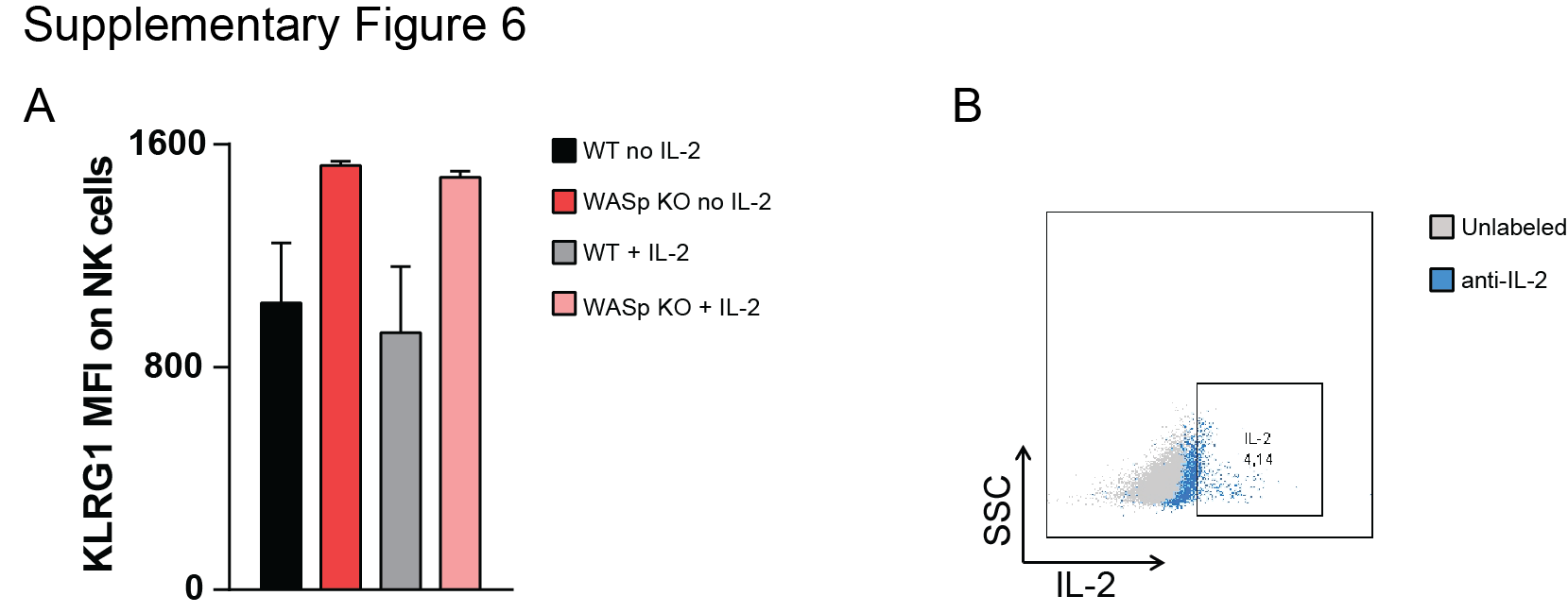


**Supplementary Fig. 6. IL-2 effect on KLRG1 expression and IL-2 secretion by A20 lymphoma cells. (A)** KLRG1 expression on WT and WASp KO C57Bl/6 NK1.1^+^CD3^-^ NK cells untreated or pre-treated with IL-2 for 48 hrs. WT n=6, WASp KO n=6. **(B)** IL-2 secretion was examined during 45 minutes in unstimulated A20 lymphoma cells. The gray dot plot represents an unlabeled control. Graphs show mean values±SEM. Abbreviations: MFI; Mean Fluorescence Intensity.
